# Transformative Frames for Climate Threat in the Anthropocene

**DOI:** 10.3389/fsoc.2021.728024

**Published:** 2021-10-29

**Authors:** Margot Hurlbert

**Affiliations:** Johnson-Shoyama Graduate School of Public Policy, University of Regina, Regina, SK, Canada

**Keywords:** transformations, frames, climate crisis, climate policy, climate emergency, climate catastrophe, anthropocene

## Abstract

This is a mini review of literature surrounding new inter and transdisciplinary frames of the threat of climate change including “Anthropocene,” linked with “climate crisis,” “climate emergency,” and “climate catastrophe”. The specific meanings and consequences of these frames are discussed and an argument why these frames are needed and risk is not enough. Ultimately, this article concludes these new framings assist transformative change by opening up climate change science, citizen engagement, and policy response. However, no one frame and no one associated policy is supported, but a plurality, dependent on context, and culture.

## Introduction

Even after the substantial impact of COVID-19, climate change- climate action failure and extreme weather-continues to be identified as the top two global risks in 2021 (WEF 2021). Climate change is occurring in all regions, and in the future more severe droughts in length and duration, as well as increases in rainfall intensity and flooding, heat stress, dry spells, and sea-level rise are expected (IPCC, 2019). As (Pelling, 2011) notes, shocks (environmental disaster, but also political and economic) manifest failure of the current social contract to provide security from disaster and offer the potential for transformative change by changing critical consciousness. Environmental changes have changed local and global discussions surrounding the “threat” of climate change increasing the use of the terms “Anthropocene,” “climate crisis,” “climate emergency,” and “climate catastrophe” (Cassegard and Thorn, 2018; Biermann and Lovbrand 2019; Jodelet, 2020; Davies et al., 2021; Ruiz-Campillo et al., 2021).

Problem framing is an important crucial precursor to efforts to drive policy change practiced by policy entrepreneurs Mintrom and Luetjens, (2017). Problem framing is constituted by highlighting failure of current policy and using interactional practices to engender changed outcomes through processes of construction and re-construction of the problem frame that draw support from multiple actors Dewulf and Bouwen, (2012). Problem framing in relation to complex problems including climate change has important implications for addressing climate change, and component problems including adaptation, mitigation, and responding to extreme events (Margot, 2018).

This article is a mini-review of literature surrounding new frames of the climate change problem including “Anthropocene,” “climate crisis,” “climate emergency,” and “climate catastrophe.” What are the specific meanings and consequences of these frames? Ultimately, do these new framings assist transformative change?.

Many argue these frames are important reflections of a new paradigm recognizing the undeniable impact humans have made on the world, ushering in a new age of ethics and recognition of a new dualism, a hybrid world of humans and nature (Cadman et al., 2021). These frames coincide with the ever narrowing window of opportunity to reduce GHG emissions and maintain global warming well below 2°C and approaching 1.5°C. Anthropocene and climate emergency authors argue these frames are a precursor for a new ethic, a longer time frame view of the world, heralding a new transformative change (Biermann and Lovbrand 2019; Davies et al., 2021).

This mini review explores these framing dynamics by first identifying the terms used in the IPCC reports and in common discourse reflected in a Google trends search. Thereafter, literature that indicates risk is not enough is followed by an examination of the emerging framing of the “Anthropocene,” “Climate Crisis,” “Emergency,” and “Catastrophe,” and a discussion of the implications of these framings. A conclusion provides insights into these framings and their implications for transformation.

## Review of Climate Risk, Anthropocene, Crisis and Catastrophe

**FIGURE 1:**
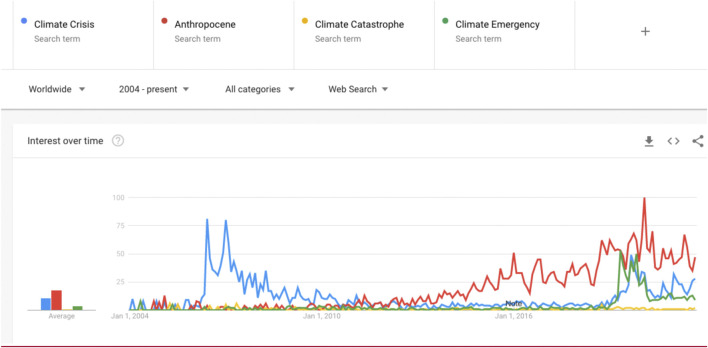
Worldwide Google trend search.

A search of the terms “Climate Crisis,” “Anthropocene,” “Climate Catastrophe,” and “Climate Emergency,” in Google trends from 2004 to date (June 2021) appears in Figure 1. This figure shows that interest in the term ‘Climate Emergency’ emerged minimally in 2009 but only increased substantively after 2018. The most significant interest has been in the term “Anthropocene” over the past 5 years followed by “Climate Crisis” and “Climate Emergency.” While “Climate Catastrophe” did appear as early as 2006 there has been relative little interest in this term.

The Intergovernmental Panel on Climate Change (IPCC) is the independent body that assesses the scientific literature and provides scientific information for addressing climate change. There isn’t a significant correlation of google trends with words used by the IPCC’s reports Wilson and Orlove, (2019).

The IPCC has organized its working groups around the key concept of risk and climate change (Reisinger et al., 2020) defining it as “the potential for adverse consequences for human or ecological systems, recognizing the diversity of values and objectives associated with such systems. (2)” The IPCC cautiously assesses, diagnosis and makes recommendations portraying climate change as a risk, describing underlying drivers and ranges of possibilities on a scientific consensus approach Weitzman, (2011). (Tubiana and Lerin, 2020) conclude the IPCC has not yet used the concept of ‘threat’ in its assessment of climate change (IPCC, 2014).

(Wilson and Orlove, 2019) document the relative absence of use of terms of “urgency,” “crisis” or “emergency” in the IPCC. The 2012 report on Extreme Events and Disasters was the high water mark using “emergency” at a rate of 21.25 occurrences per 100,000 words. However, this use has declined never rising above a rate of 5 for “threat” terms in subsequent reports (ibid.). Signaling a need for closer scrutiny of more “emergent” frames, climate experts at COP18 in Doha warned that the IPCC was being overly conservative, underestimating climate change impacts, and in fact the worst case scenario of AR4 had been realized (Tubiana and Lerin, 2020). Questions arise in understanding the meaning and genesis of these threat frames.

### Indications that Climate Risk is Not Enough

(Beck, 2016) argued in his posthumously published book that because of the immense threat of environmental crises, we are living in time of a metamorphosis. The challenge is to create a new cosmopolitan order as the current international order is not able to tackle the issue of the climate catastrophe. Moving from the concept of “risk” to “threat” is documented by Jodelet, (2020) to be increasingly predominate and altering representations and sensitivities. While risks are often adopted as rational assessments, threats conceptually move beyond risks to include dimensions that are subjective and emotional and recalled for a period long expired. (Bourg, 2020) identifies a category of “transcendental damage” that threat implies that provokes the collapse of humankind itself, the very habitability of the Earth System for humans. Consistent with (Rimé, 2020), threats drive reactions including search for information, overabundant conversations, crowd gatherings and manifestations of generosity and openness to others. These function to reweave or recreate a social fabric, reaffirming common values and beliefs, restoring solidarity and social cohesion.

The shift of conceiving of climate change as a threat- a catastrophe- instead of a risk is documented between the COP in Copenhagen and Paris by Tubiana and Lerin (2020). While statements in Copenhagen focused on differentiated responsibility, justice, finance (President Obama’s speech focused on transparency, financing and design of the review mechanism), in Paris statements focused on the current impacts of climate change. President Obama’s speech focused on dramatic experienced climate impacts in Alaska as did many other world leaders. Although the IPCC’s hadn’t raised the threat issue, because world leaders focused on the omnipresent threat of climate change at the Paris COP, (Tubiana and Lerin, 2020) identify the link this made to the important action that was achieved in the Paris agreement.

### The Anthropocene

Many scholars are arguing we have arrived at a new geological epoch, the “Anthropocene,” including geologists (Zalasiewicz Waters and Summerhayes, 2017), political scientists (Biermann and Lovbrand, 2019), legal scholars, (Cadman et al., 2021), criminal justice scholars (Shearing, 2015), and philosophers (Arias-Maldonado, 2019). In this new epoch, humans are credited with having produced a stratigraphic signature in sediments and ice distinct from the Holocene epoch (Waters et al., 2016). The word *anthropos* (Ancient Greek for “human”) informs this title (ZalasiewiczWaters and Summerhayes, 2017). This word defines the epoch by humans’ intractable impact on the Earth including contribution to the Earth’s sixth extinction, causation of radioactive fallout embedded in Earth’s sediments since the 1940s, and demand for Earth’s resource making the broiler chicken the largest standing stock of any other bird species on the plant (estimated at 22.7 billion in 2016) (Bennett and Thomas, 2018). The idea of the Anthropocene does have critics for overemphasizing human mastery or erasing differential human responsibilities including imperialism and capitalism, racism (Mathews, 2000) and glossing over the differences between the developed world that is responsible for causing climate change and the Global South that is the most vulnerable to its consequences (Crist, 2013). For other scientists, the epistemic, metaphoric, and narrative potential of this designation has caught their attention (Shearing, 2015; Biermann and Lovbrand, 2019; Cadman et al., 2021).

Other paradigms have challenged traditional human versus nature (Human Exemptionalism Paradigm) and Judeo-Christian hierarchies of man and animals including Dunlop’s New Ecological Paradigm (Dunlap et al., 2000). Disaster and risk framings have existed for many decades, if not centuries, but the Anthropocene term changes thinking to embrace large time scales and surpass traditional gridlocked thinking that has resulted in failures of futurological imagination (Ialenti, 2020).

Global environmental change is now recognized as the direct result of human agency. Not only is the human the actor responsible for social relations including global geopolitical and socioeconomic change, but the human is a biophysical “actant” reshaping the earth through actions−a biophysical being that interacts and co-determines other biophysical beings (Shearing, 2015). The term the “Anthropocene” captures the scale and complexity of our planet’s health where terms such as “nature” and “environment” have failed (Biermann and Lovbrand, 2019). The term “Anthropocene” embodies an enlightenment tradition that moves beyond mechanistic traditional socio-environmental studies to a lively and vibrant reflection on inter-relations that are in constant transformation (Arias-Maldonado, 2019). In addition to advancing the study of new forms of power (between peoples, and people with and for the planet) the Anthropocene implies a new sense of urgency, an unease about humanity’s role in the world (as planetary boundaries are breached), and calls for new ethical debates (Biermann and Lovbrand, 2019).

These debates have also led to a re-conception of democracy in the Anthropocene. In this re-conception, decision making moves beyond involving people in developing new policies and institutions, but appointing amicus curie to represent inanimate actors (including rivers, the trees, the planet, etc.) in decision making (Mert, 2019). In the Anthropocene not only is decision-making participatory and transparent, but so is knowledge making. In the previous Holocene, while knowledge was co-constructed between people and scientists, in the Anthropocene knowledge creation is set free. In this analytical space cultural and social assumptions are debated and scientist and people collectively make sense of change in inter and transdisciplinary ways (Strohschneider and Visbeck 2016; Beck, 2019). By creating an interdisciplinary imaginary of the “Anthropocene” multiple disciplines tackle the science issues of the Anthropocene using multiple approaches, different power dimensions, new ethical debates, new forms of decision making, and new relations of scientists and people making change.

### The Climate Crisis, Emergency, and Catastrophe

The Anthropocene implies a new sense of urgency (Biermann and Lovbrand, 2019) that links directly with a largely coincidental emergency formulation of climate change. The development of climate change crisis has occurred within at least the last decade (Olsson, 2009); crisis is defined as an event or process that is uncertain, but constitutes a threat that is urgent in relation to core community structures and values (Boin et al., 2017). While the use of the word “crisis” spiked in 2006 with Al Gore’s release of “An Inconvenient Truth,” it once again spiked after 2017. The more recent emergence coincided with the use of the related term “climate emergency” (Wilson and Orlove, 2019) (see Figure 1). (Wilson and Orlove, 2019) point out the term “crisis” is “one of the most illusive, vague, imprecise, malleable, open-ended and generally unspecified concepts” (3). While both crisis and emergency describe a broad range of threats or negative situations, an “emergency” introduces properties of immediacy and danger (Markusson et al., 2014), but contains seeds of emancipatory potential (Anderson, 2017), and arguably exists within the already existing framing of emergency response and planning. Crisis could imply failure at multiple levels that include government actors (Boin and Lodge, 2016). While activists interchange use of the term crisis and emergency, nearly 1,500 governments worldwide have declared a climate emergency (Ruiz-Campillo et al., 2021).

Several authors argue that climate emergencies do not go far enough and need to endorse and specify urgent change (Ripple et al., 2021). Others identify that this language may be misused. In Ireland a climate emergency declaration was accompanied by a “money message” policy tool attached to the Climate Emergency Measures Bill. The bill delayed any action as it raised implications for public finances (Fitzgerald et al., 2021). O’Neill and Sinden (2021) documented how universities’ declarations coincided with strategies of market differentiation, sustainability capital and competition for students.

In some contexts, the crisis and emergency framing has been extended to a “catastrophic” framing. Cassegard and Thorn (2018) argue we are witnessing the emergency of a “post-apocalyptic environmentalism”. Activists including Fridays for Future and Extinction Rebellion represent demands for climate action based on the view the crisis is beyond a point where it can be solved, or that we have reached a point of catastrophic loss that has already occurred and is impossible to prevent. This framing is captured by such titles as The Uninhabitable Earth (Wallace-Wells, 2017) and ‘We’re doomed. Now What? (Scranton, 2018). Cassegard and Thorn (2018) argue these descriptions may sound defeatist, but produce hope through acceptance of loss and engendering an imagination of what is possible after the apocalypse. In the words of Jem Bendell, 2019 of Extinction Rebellion:

Bold emissions cuts and carbon drawdown measures are still necessary to reduce as much as possible the mass extinction and human suffering of climate change, but we must also prepare for what is now inevitable … as we no longer assume that society as we know it can continue (2019).

## Framing Implications: Securitization, and Mobilization for Transformation

Issues raised with the emergency framing relate to concern about implying a deadline in relation to a short and closing window (Asayama et al., 2019) or triggering a new “state of exception” manifestation that legitimizes new forms of authoritarianism (Davies, 2019) not necessarily overcoming the challenges of stimulating ambitious climate action or overcoming the legitimacy of emergency as strategy planning (Patterson et al., 2021). However, in a study of 300 declarations from local governments of climate emergencies, Ruiz-Campillo et al. (2021) concluded that they did not engender the “state of exception” concern. Instead, these declarations were performative acts signaling policy changes aligning the operation of local government with the stated motivations of integrating climate change in planning, impact assessment, policy making. The declarations also redefined local governance by opening the door to dialogue across local government, with social movements and the private sector. Much literature documents governments, non-profit and research driven engagement arising from the climate crisis and emergency language including Edinburgh’s Climate Commission (Creasy et al., 2021), citizen assemblies (Devaney et al., 2020; Sandover et al., 2021), and efforts to connect with vulnerable groups using an emancipatory and intersectional lens (Davies and Hugel, 2021; Kenis, 2021; Stripple et al., 2021).

However, in contrast to (Cassegard and Thorn, 2018) above, affective response to “catastrophe” messaging must be considered, including worry, helplessness, disgust, fear, rage etc. that may result in maladaptive decisions (Ruiter et al., 2004). Framings of crises often do not engender a sense of empowerment, and there is much we don’t know about time pressure messaging, cognition, motivation to act, personal levels of knowledge, and decision-making heuristics; but we do know that people cognizant of greater climate change risks, who are better informed, are more likely to support climate change policies (Wilson and Orlove, 2019). Wilson and Orlove (2019) conclude that framing climate change as a crisis without providing accompanying information about self-efficacy (the ability to make change) and hope, will likely polarize people and their beliefs and actions.

In addition to individual affective responses, country context or culture is also important. In order to enter into a dialogue surrounding climate change threat, understanding different countries and peoples’ views, climate change policies, and ecological practices is fundamental (Caillaud and BonnotKrauth-Gruber, 2020). Emotional dimensions and pre-existing beliefs are necessary to tailor public campaigns to raise awareness and change behaviour. National and personal identity are central. For instance, in one such exploratory study, qualitative research studies in Germany found people were predominately altruistic (other-centered) while results in France concluded participants were self-centered (Caillaud and BonnotKrauth-Gruber, 2020). This knowledge will be important for framing policy response to the climate change problem framing.

For instance, (Hulme, 2019) argues that the framing “net-zero carbon emissions” as a target is narrow and sets unhelpful policy goals both insufficiently ambitious and crowding out other concerns. However, currently, two countries have achieved net zero emissions, six countries have passed legislation to achieve net zero emissions, six more countries have proposed legislation, and many more are in policy document or under discussion (Energy and Climate Intelligence Unit, 2021). An alternative framing offered by Hulme would focus on Sustainable Development Goals (SDGs), which is also recognized as important policy framing IPCC, (2018). For transformations, climate change framing literature supports the co-existence of multiple policy instruments and the importance of a complete policy suite (Margot, 2018; IPCC 2019). Consistent with Scoones et al. (2020), plural pathways to achieve transformations that entail different ideas and values imply multiple, very material, transformations that engage across new forms of deliberation, diverse ideas, contrasting norms, interests, practices and actors.

In a similar vein, it is important that knowledge production in the Anthropocene not be narrowed. The Anthropocene arguably engenders an opening of climate science, consistent with transformations that advance plural knowledge systems, including Indigenous and local knowledge (Lam et al., 2020) and knowledge expansion or greater time, spatial, and social scales, rejecting technocratic closure (Few et al., 2017). If narrowing and limiting social and policy problems occurs, then there will be a narrowing of climate projects; if there is only tokenistic participation there is risk of mere “performative pathways”. It may be that unpredictable and unique knowledge creates the pathway of transformation (Beck, 2019). The Anthropocene offers the possibility of achieving true inter and transdisciplinary science, and interrogating the assumptions and values of science to a range of social concerns and meanings. By opening up science, new conditions for knowledge formation and alternative directions for public agendas and policies are allowed Blue and Medlock, (2014).

## Conclusion

As the impacts of climate change accelerate and the window of opportunity closes for making transformative changes to address climate change, there has been an increasing use of the “threat” frames including “Anthropocene,” “climate crisis,” “climate emergency,” and “climate catastrophe” (Cassegard and Thorn, 2018; Biermann and Lovbrand 2019; Jodelet, 2020; Davies et al., 2021; Ruiz-Campillo et al., 2021). And while all terms advance policy changes, they have specific meanings and consequences. It is advisable that their use, by whom, and in what circumstance, is mindful of subtle differentiation. This is not to say that any of these terms should be embargoed. However, depending on the actor and situation, one or another may be more appropriate.

It is clear that risk frames are not enough to engender the transformative change required to address climate change (*The Anthropocene*). Thus, a case is made for these new framings. Transformative climate science is defined as an “open-ended process of producing, structuring, and applying solutions-oriented knowledge to fast-link integrated adaptation and mitigation strategies to sustainable development” (David Tàbara et al., 2018: 807). This mini review has documented successful use of these terms, reasons for caution surrounding “Climate Crisis” and “Climate Catastrophe,” but important connections with “Climate Emergency,” the most efficacious frame linked with innovative policy change. Coincident with support for more research and understanding of the use of a combination of these terms, is support for plural policy responding to these frames. For transformative changes, context, culture, and geography matter.
